# Innovations in Treating Headache

**DOI:** 10.3390/life15121792

**Published:** 2025-11-21

**Authors:** Barnabas T. Shiferaw, Muhammed Zahid Sahin, Alaa Abd-Elsayed

**Affiliations:** 1 Department of Anesthesiology, University of Wisconsin School of Medicine and Public Health, 600 Highland Avenue, B6/319 CSC, Madison, WI 53792-3272, USA; bshiferaw@wisc.edu; 2 Department of Physical Medicine and Rehabilitation, Sakarya University Training and Research Hospital, Faculty of Medicine, Sakarya University, 53705 Sakarya, Türkiye; zsahin@sakarya.edu.tr

Headache represents one of the most frequent neurological complaints encountered in daily clinical practice and remains a leading cause of disability worldwide. Most cases fall under primary headache disorders such as migraine, tension-type headache, and trigeminal autonomic cephalalgias, while secondary headaches arise from structural, infectious, or vascular causes [1]. Conventional management typically involves lifestyle modification, acute and preventive pharmacological treatments, and behavioral strategies. Despite these well-established approaches, a substantial proportion of patients continue to experience unsatisfactory relief due to incomplete efficacy, adverse effects, or contraindications to take the prescribed drugs. This therapeutic gap has prompted increasing interest in interventions for pain control that offer targeted modulation of pain pathways. Techniques such as corticosteroid injections, peripheral nerve stimulation, localized nerve blocks, and radiofrequency ablation (RFA) are gaining recognition as effective adjunctive or alternative options, reflecting a broader shift toward individualized, multimodal headache care [2].

Peripheral nerve stimulation (PNS) has become one of the most extensively investigated neuromodulation strategies. Introduced in the late 1960s and refined with less invasive percutaneous techniques in the early 2000s, PNS modulates nociceptive transmission by altering frequency, pulse width, and intensity of sensory fiber activity [1,3]. Occipital nerve stimulation (ONS), in particular, has been studied in the treatment of chronic cluster headache, demonstrating meaningful reductions in attack frequency; however, complications such as lead migration or hardware malfunction remain challenges [1]. Vagus nerve stimulation (VNS) has similarly evolved, with noninvasive cervical VNS receiving FDA approval for the treatment of migraines in 2018, and auricular VNS showing particular promise in reducing headache days [4]. External trigeminal nerve stimulation (eTNS) further expands the spectrum, with sham-controlled trials reporting reduced migraine days and medication use, without significant side effects [1]. These advances highlight a shift toward safer, more accessible neuromodulation, while invasive options remain crucial for highly refractory cases.

Nerve blocks also remain essential, offering diagnostic clarity and short-term relief. Blocks of the third occipital, greater and lesser occipital, and facial nerves can reduce pain and associated symptoms, though benefits are usually temporary. The sphenopalatine ganglion (SPG) is another important target, especially in cluster headache, where topical and injection-based techniques have demonstrated benefit [2].

Beyond peripheral interventions, noninvasive cortical stimulation offers another promising approach. Repetitive transcranial magnetic stimulation (rTMS) and direct current stimulation (tDCS) modulate cortical excitability and pain networks. A network meta-analysis found that both rTMS and tDCS reduce migraine frequency and medication use, with tDCS appearing more effective than rTMS or VNS for pain intensity and attack frequency [5]. Both methods are generally safe, well-tolerated, and potentially cost-effective, though standardized protocols and large confirmatory trials remain essential.

Despite advances in less invasive methods, invasive techniques still play a minor but essential role in resistant cases. Deep brain stimulation of the posterior hypothalamus can reduce cluster headache attacks in highly refractory patients; however, its risks and high cost limit its use to specialized centers [6]. Cervical spinal cord stimulation may also help alleviate chronic intractable headaches, although current evidence is still preliminary [7].

Expanding the therapeutic spectrum, RFA provides a bridge between noninvasive neuromodulation and surgical interventions. RFA is increasingly considered a valuable option for the treatment of chronic headaches [8]. Studies have shown that pulsed RF (PRF) provides higher patient satisfaction compared to steroid injections. Ablation of the C2 and third occipital nerves is also associated with a high willingness to repeat treatment if pain recurs, reflecting strong patient satisfaction [9]. Prospective data on the sphenopalatine ganglion indicate that RFA and PRF offer similar benefits, with a duration of approximately five months [10]. Consistent patient satisfaction underscores RFA’s potential to provide meaningful and durable relief.

Building on these advances, innovative ablative procedures are further expanding treatment options. One such innovation is the ‘ALblation’ technique, developed by Alaa Abd-Elsayed for patients with bilateral chronic migraine resistant to medications. The technique involves sequential lesion-mode RFA targeting the supraorbital, supratrochlear, greater occipital, and lesser occipital nerves bilaterally, following diagnostic and confirmatory nerve blocks. Early clinical experience has demonstrated substantial and sustained reductions in headache intensity and frequency, with benefits maintained in the long term and no significant adverse effects reported [11]. These findings suggest that targeted peripheral ablation may help disrupt central sensitization and provide durable relief in patients who are otherwise difficult to treat. The technique’s minimally invasive nature and favorable safety profile make it a practical option for outpatient use and repeat procedures if symptoms recur.

Beyond current applications, targeted peripheral ablation holds promise for broader integration into multimodal pain management strategies. Combining ablative therapy with noninvasive neuromodulation, such as transcranial stimulation or VNS, may enhance outcomes through synergistic modulation of central and peripheral pain pathways.

These advances signal that headache management is entering an era of innovation. Noninvasive neuromodulation methods such as eTNS, auricular VNS, and tDCS provide safe, accessible options for patients who cannot tolerate medications. At the same time, invasive approaches such as ONS, DBS, and RFA remain necessary for treating refractory cases. Newer techniques such as ALblation show how interventional strategies are becoming more precise and effective. Future progress will rely on refining protocols, confirming results through larger studies, and ensuring the cost-effectiveness of these approaches.

Looking ahead, the continued evolution of interventional headache management will rely on close interdisciplinary collaboration among neurologists, pain specialists, and rehabilitation physicians. Establishing standardized training programs and evidence-based procedural guidelines will be crucial to ensuring safety, optimizing outcomes, and expanding access to these therapies.

## Abbreviations

The following abbreviations are used in this manuscript:

PNS	Peripheral Nerve Stimulation
ONS	Occipital Nerve Stimulation
VNS	Vagus Nerve Stimulation
eTNS	External Trigeminal Nerve Stimulation
SPG	Sphenopalatine Ganglion
rTMS	Repetitive Transcranial Magnetic Stimulation
tDCS	Transcranial Direct Current Stimulation
DBS	Deep Brain Stimulation
RFA	Radiofrequency Ablation
PRF	Pulsed Radiofrequency
FDA	Food and Drug Administration
